# A Photosynthetic Light Acclimation Model Accounting for the Effects of Leaf Age, Chlorophyll Content, and Intra-Leaf Radiation Transfer

**DOI:** 10.3389/fpls.2022.889709

**Published:** 2022-06-22

**Authors:** Jan Graefe, Wenjuan Yu, Oliver Körner

**Affiliations:** ^1^Leibniz-Institute of Vegetable and Ornamental Crops (IGZ), Next-Generation Horticultural Systems, Grossbeeren, Germany; ^2^Department of Functional Genome and Gene Safety, Chinese Academy of Agricultural Sciences, Beijing, China

**Keywords:** light acclimation, *J*_max_, chlorophyll, tomato, intra-leaf, age, *LMA*, *V*_cmax_

## Abstract

Mechanistic models of canopy photosynthesis usually upscale leaf photosynthesis to crop level. A detailed prediction of canopy microclimate with accurate leaf morphological and physiological model parameters is the pre-requisite for accurate predictions. It is well established that certain leaf model parameters (*V*_cmax_, *J*_max_) of the frequently adopted Farquhar and Caemmerer photosynthesis model change with leaf age and light interception history. Previous approaches to predict *V*_cmax_ and *J*_max_ focused primarily on light interception, either by cumulative intercepted photosynthetic photon flux density (PPFD) or by closely related proxy variables such as leaf nitrogen content per leaf area. However, for plants with monopodial growth, such as vertically grown tomatoes or cucumber crops, in greenhouse production, there is a strong relationship between leaf age and light interception, complicating the experimental and mathematical separation of both effects. We propose a modeling framework that separates age and light intensity-related acclimation effects in a crop stand: Improved approximation of intra-leaf light absorption profiles with cumulative chlorophyll content (*Chl*) is the basis, while parameters are estimated *via* Gaussian process regression from total *Chl*, carotenoid content (*Car*), and leaf mass per area (*LMA*). The model approximates light absorption profiles within a leaf and links them to leaf capacity profiles of photosynthetic electron transport. Published datasets for *Spinacia oleracea* and *Eucalyptus pauciflora* were used to parameterize the relationship between light and capacity profiles and to set the curvature parameter of electron transport rate described by a non-rectangular hyperbola on *Cucumis sativus*. Using the modified capacity and light absorption profile functions, the new model was then able to predict light acclimation in a 2-month period of a fully grown tomato crop. An age-dependent lower limit of the electron transport capacity per unit *Chl* was essential in order to capture the decline of *V*_cmax_ and *J*_max_ over time and space of the investigated tomato crop. We detected that current leaf photosynthetic capacity in tomato is highly affected by intercepted light-sum of 3–5 previous days.

## Introduction

At the heart of most experimental and theoretical plant growth studies are measurements or predictions of primary CO_2_ assimilation at different spatial and temporal scales. Mathematical or biological integration of instantaneous CO_2_ assimilation rates over total leaf area and day/night cycle cumulates to daily biomass growth rates excluding certain losses. Therefore, there has been much work on modeling leaf photosynthesis (von Caemmerer et al., 2009), canopy microclimate (Russell et al., 1990; Körner et al., 2007; Myneni and Ross, 2012), and its proper integration (Bonan et al., 2021) over the last decades. In addition, mechanistic models of canopy photosynthesis require for upscaling from leaf photosynthesis rates an accurate description of microclimate and well-estimated leaf-model parameters at different canopy positions.

Certain parameters (e.g., *V*_cmax_ and *J*_max_) of the frequently used Farquhar–Caemmerer–Berry (FCB) leaf photosynthesis model (von Caemmerer et al., 2009) are not constant over time and change with leaf age and past light interception. Photosynthetic acclimation to shade is a well-investigated process both at leaf (Lichtenthaler and Babani, 2004) and intra-leaf levels (Nishio et al., 1993). Focus was often set on light acclimation using either the cumulative intercepted photosynthetic photon flux density (PPFD) or closely related proxy variables, such as the leaf nitrogen content per leaf area, as predictors for *V*_cmax_ and *J*_max_ (Meir et al., 2002; Niinemets et al., 2004).

For plants with a monopodial growth habit, such as vertically grown tomatoes or cucumber crops, in greenhouse production (as common in commercial practice), there is a strong relationship between leaf age and light interception (Niinemets, 2016), complicating the experimental and mathematical separation of both effects. This may limit the generality of previously developed acclimation models, especially with the introduction of novel cultivation procedures, e.g., intra-canopy lighting (Joshi et al., 2019). To prevent the concurrent change of leaf age and intercepted light, plants could be grown horizontally (Trouwborst et al., 2011a). This, however, is unpractical and introduces artifacts, e.g., the vertical dominance among plant organs is disturbed.

In this article, we hypothesized that modeling light and age acclimation at the intra-leaf level is a feasible approach for estimating vertical parameter profiles over time, i.e., it enables the separation of age and light intensity-related effects in a crop stand. Besides reanalyzing several datasets from the literature, we performed a greenhouse experiment with a vertical growing tomato crop observing leaf parameters in different canopy depths over time. From that, we assessed the spatial-temporal evolution of *V*_cmax_ and *J*_max_.

## Materials and Methods

### Model Theory, Extension, and Parameter Estimation

#### Light Absorption Profiles Within a Leaf

The intra-leaf profile of incident and absorbed radiation can be well described by a two-stream-type approach of simultaneous downward and upward radiation transfer with cumulative chlorophyll (a + b) content *c* within the leaf mesophyll (Terashima et al., 2009). The absorbed light intensity *I*_*a*_(c) from both streams can be approximated by a simple exponential profile of incident light *I*(c) times a two-stream absorption coefficient *k*_*a*_ (Badeck, 1995; Buckley and Farquhar, 2004).


(1) $$I_{a}\,\left(c\right)=k_{a}\,I\,\left(c\right)=I_{0\,}\,p_{1}\,k_{a}\,\exp\left(-k\,c^{p_{2}}\right)$$

With incident irradiance *I*_0_ on the upper leaf side, effective extinction coefficient *k*, scaling parameter *p*_1_, and exponent *p_2_.* We introduced the exponent *p*_2_ to allow for an improved fit of Equation 1 to the two-stream solution.

As neither in nature nor in experimental systems, light incidence is exclusively one-sided, Equation 1 was generalized for a two-sided incidence by Buckley and Farquhar (2004) as follows:


(2) Ia(c,wu,I0,k)=I0⁢p1ka (wu exp (-kcp2)+(1-wu) exp (-k(Chl-c)p2))

with total chlorophyll content (*Chl*) per leaf area [c = (0, *Chl*)] and fractional light incidence *w*_*u*_ on the upper leaf side, where *I*_0_ here denotes the total incident light on both leaf sides.

To obtain predictive equations for the introduced parameters (*p*_1_, *p*_2_, *k*_*a*_, and *k*), we applied the *Prospect-D* leaf spectra model (Féret et al., 2017) and computed scattering and absorption coefficients (*k*_*s*_ and *k*_*a*_) with a two-stream solution within the leaf mesophyll:


(3) $$\begin{array}{cc} \frac{d\,I_{d}}{d\,c}=-\left(k_{s}+k_{a}\right)\,I_{d}+k_{s}\,I_{u} & w\,i\,t\,h\;I_{d}\,\left(0\right)=\left(1-r_{e}\right)+r_{i\,}\,I_{u}\,\left(0\right)\\ \frac{d\,I_{u}}{d\,c}=\;\left(k_{s}+k_{a}\right)I_{u}-k_{s}I_{d} & \,I_{u}\,\left(C\,h\,l\right)=r_{i\,}\,I_{d}\,\left(C\,h\,l\right) \end{array}$$

with downward and upward propagating diffuse radiation fluxes *I*_*d*_ and *I*_*u*_, respectively. External *r*_*e*_ (air → epidermis) and internal leaf surface reflectance *r*_*i*_ (epidermis → air) are calculated from leaf spectral refraction index (*n*, Féret et al., 2017) and by solving the Fresnel equations for diffuse incident light (Stern, 1964; Jacquemoud and Baret, 1990). The general solution of Equation 3 was obtained (refer to Jacquemoud and Ustin, 2019) with two free constants (C_1_ and C_2_) to be estimated from boundary conditions stated in Equation 3. With given total leaf reflectance and total transmittance (*R*, *T*),


(4) $$\begin{array}{cc} R= & I_{u}\,\left(0,{\text{C}}_{1},C_{2}\right)\,\left(1-r_{i}\right)+r_{e}\,\\ T= & I_{d}\,\left(C\,h\,l,C_{1},C_{2}\right)\,\left(1-r_{i}\right) \end{array}$$

The radiation transfer parameters *k*_*s*_ and *k*_*a*_ are estimated from the solution of Equation 4, and the forward problem (Equation 3) to obtain *I*_*d*_(*c*) and *I*_*u*_(*c*) can be computed.

#### Parameter Estimation of Light Profile Function

In our approach, *I*_*a*_(c) was subsequently parameterized (i.e., *p*_1_, *p*_2_, *k*_*a*_, and *k*) by a five-step procedure using leaf *Chl*, leaf carotenoid content (*Car*), and leaf mass per area (*LMA*):

1. A set of 470 leaves from the Lopex and Angers leaf spectral dataset (Jacquemoud et al., 2003) were selected (i.e., selected leaves exceed the 5% percentile values of *Chl* and leaf mass water content over the whole dataset).
2. Solving Equations 3, 4 for those leaves resulted accordingly in *i* = 1…470 values for *k*_*s,i*_ *k*_*a,i*_ and corresponding profiles of incident radiation *I*(c) = *I*_*d,i*_(c) + *I*_*u,i*_(c).
3. The obtained spectral values of *I*_*d,i*_,(c, λ) + *I*_*u,i*_(c, λ) between 400 and 700 nm were integrated according to a D55 CIE daylight spectral density distribution (Muschaweck, 2021) characterizing a typical daytime sky. The two-stream spectral absorption coefficients *k*_*a*_(λ) were combined similarly to spectral light intensities. In addition, photosynthetic effective absorption (i.e., assuming 100% for chlorophylls and 70% for carotenoids; Laisk et al., 2014) was accounted for by using the absorption spectra for chlorophyll, carotenoid, leaf dry matter, and water from the Prospect D model.
4. Spectral integrated *I*_*d,i*_(c) + *I*_*u,i*_(c) were then used to fit *p*_1_, *p*_2_, and *k* in Equation 1.
5. All obtained parameter sets (*p*_1_, *k*_*a*_, *k*, *n* = 470) were analyzed *via* machine learning (Gaussian process regression, MATLAB R2020a, Regression Learner App) using the leaf parameters (features), namely, *Chl*, *Car*, and *LMA*.

#### Modeling Photosynthetic Electron Transport

To estimate the whole leaf electron transport rate *J*_*leaf*_, electron transport rate per unit chlorophyll *J*_*c*_(*c*) is integrated over cumulative *Chl* (i.e., mesophyll thickness; Badeck, 1995; Buckley and Farquhar, 2004) using the Blackman response (linear slope and asymptote, Equation 5). This is a good approximation for the light response of electron transport rate at single cell or chloroplast level (Terashima and Saeki, 1985):


(5) $$J_{l\,e\,a\,f}\,\left(I_{0},\,C\,h\,l\right)=\int_{0}^{C\,h\,l}J_{\text{c}}\,\left(c\right)\,\mathrm{dc}=\min\,\left[\varphi \,\,I_{a}\,\left(c,\,w_{u,m},I_{0},k\right),J_{c,m\,a\,x}\,\left(c,w_{u,g},\,I_{*},k^{{}^{\prime }}\right)\,\right]\,d\,c$$

with PSII quantum efficiency of electron transport φ, fractional upper light incidence during measurement *w*_*u,m*_ and growth *w*_*u,g*_, respectively, a modified extinction coefficient *k*’, characteristic leaf irradiance I* during light acclimation (Buckley and Farquhar, 2004), and maximum electron transport rate per unit chlorophyll *J*_c,max_. As a generalization of Equation 5, we apply a non-rectangular hyperbola with curvature parameter θ for *J*_*c*_(*c*) with the equation as follows:


(6) $$J_{c}\,\left(c\right)=\,\left(\varphi \,I_{a}+J_{c,m\,a\,x}-\sqrt{{\left(\varphi \,I_{a}+J_{c,m\,a\,x}\right)}^{2}-4\,\theta \,\varphi \,I_{a}\,J_{c,m\,a\,x}}\right)/\left(2\,\theta \right)$$

Following Buckley and Farquhar (2004), *J*_*c,max*_ is described as a function of absorbed radiation profile with a characteristic light intensity I*. We adopted that approach and extended it in three ways, namely, (1) time-dependent minimum [*J*_*c,max,mn*_(t)] and (2) maximum [*J*_*c,max,mx*_(t)] values, respectively, and (3) a modified extinction coefficient *k*′ = *p*_3_k (Equation 7). With *p*_3_ = 1, the capacity profile of electron transfer would match the light absorption profile perfectly.


(7) Jc,m⁢a⁢x(c,wu,g,I*,k)′=min{Jc,m⁢a⁢x,m⁢x(t),max[Jc,m⁢a⁢x,m⁢n(t),φIa(c,wu,g,I*,k)′ ]}

The characteristic light intensity I* is determined from the light intensity history (i.e., past days) of each specific leaf. Besides light-induced changes in *Chl*, *Car*, and *LMA*, which determine the intra-leaf profiles (*k*′) and optical depth (*Chl*), I* may be interpreted as a mathematical proxy for light-induced changes of key photosynthetic enzymes or complexes (e.g. cytochrome b_6_*f*) to chlorophyll ratios (Evans and Seemann, 1989; Eichelmann et al., 2005; Schöttler and Tóth, 2014).

Equation 2 may be applied to leaf gas exchange measurements obtained from a cuvette system (e.g., LI-6400, LICOR Bioscience) with an actinic light source at one leaf side. For that, leaf transmittance needs to be taken into account. Denoting the reflectance of the lower chamber wall by *r*_*ch*_ and neglecting multiple reflections, one obtains w_*u,m*_ = 1/(1 + *T*⋅*r*_*ch*_) and *I*_0_’ = *I*_0_(1 + *T⋅r_*ch*_*). Total leaf transmittance *T* is also estimated from *Chl*, *Car*, and *LMA* using Gaussian process regression. For the LI-6400 standard lower chamber wall, we assumed *r*_*ch*_ = 0.5. The quantum efficiency φ of absorbed photons was estimated using an expression given by Yin et al. (2004)


(8) $$\varphi =\,\frac{1-f_{c\,y\,c}}{1+\left(1-f_{c\,y\,c}\right)/{\text{Φ}}_{2\,m}}$$

With assumed values for the fraction of cyclic electron flow *f*_*cyc*_ (0) and maximum e^–^ transport efficiency of PSII Φ_2m_ [0.88, refer to discussion in Kalaji et al. (2017)]. Equation 8 yields φ = 0.468. Other effects of leaf absorptance α_*L*_ and non-photosynthetic contributions *f* are fully accounted for by *I*_*a*_ (Equation 2). This is similar to the approach frequently used for bulk leaves (von Caemmerer et al., 2009)


(9) φ=′φαL(1-f)

Mathematically, *J*_max_ is the integral of *J*_*c,max*_(c) over the cumulative *Chl*, but in the context of A/C_*i*_ curves, the retrieved *J*_max_ should be rather approximated as *J*_*leaf*_(*I*_0_′, *Chl*) at constant light intensity *I*_0_. Assuming a unique proportionality between the capacities of electron transport and the Calvin cycle throughout the leaf, *V*_cmax_ is given by


(10) $$V_{c\,m\,a\,x}=p_{4}\,\int_{0}^{C\,h\,l}J_{c,m\,a\,x}\,\left(c,w_{u,g},\,I_{*},k^{{}^{\prime }}\right)\,d\,c$$

with additional parameter *p*_4_.

### Empirical Data

#### Tomato Greenhouse Experiment

##### Experiment and Crop Management

Tomato seeds (*“Pannovy”*) were sown on 2 January 2018; 9 days after sowing, 48 seedlings were transplanted to stone-wool cubes and placed in a greenhouse controlled at 18°C at the Leibniz Institute of Vegetable and Ornamental Crops (IGZ), Großbeeren, Germany (52.35 N 13.31 E). On 22 February 2018, 48 tomato plants were selected by uniformity and placed on inert fleece mats with drip irrigation in four rows of each 12 plants in one central compartment (28.8 m^2^) of the gas-exchange greenhouse (GEGH) at the IGZ (Kläring and Körner, 2020). The remaining seven compartments were equipped in the same way, i.e., border effects were minimized. For a starting period of 12 days, the temperature was controlled to 19°C and 15°C during day and night, respectively; air relative humidity (RH) was set to 80% and air CO_2_ concentration was maintained at 400 μmol mol^–1^ during daytime. From 5 March 2018, the greenhouse temperature was set at 23°C, while all other setpoints remained unchanged. During all time, water and nutrients were adequately supplied by an automated non-recirculating system. The nutrient solutions were prepared after de Kreij et al. (2003) and were adjusted daily to constrain electric conductivity (EC) between 2.2 and 2.5 dS m^–1^ and to a mean pH of 5.6. The canopy was maintained at 4 m heights, and the mean leaf number was 18 leaves per plant (counting leaves > 10 cm in length).

##### Measurements and Computations

Each plant in the canopy was virtually subdivided into 8 vertical layers. For a leaf residing in layer *i*, the overlaying *Leaf Area Index* counted to the top (*LAI*_*t,i*_) was estimated from $L\,A\,I_{t,i}=\left(\sum_{1}^{i}2\,S_{L,j}+S_{L,i}\right)/S_{p}$ with total ground area per plant *S*_*P*_ (4,167 cm^2^) and one-sided leaf surface area *S*_*L,j*_ (cm^2^) in layer *j*. Note that one of the two leaves is included in target layer *i*. The area of a single leaf was derived from time-dependent length (*L*) and width (*W*) of leaves as S_*L*,*i*_ = 0.2568⋅W(*t*_*L*_) ⋅ *L*(*t*_*L*_) + 11.725 where leaf age (*t*_*L*_) dependence was adopted from Yu and Körner (2020).

Using hourly recorded air temperatures from a within canopy-installed psychrometer, we calculated the effective thermal time for tomato phenology using a response function with cardinal temperatures adopted from the CROPGRO-Tomato model (Boote et al., 2012). Outside the greenhouse, recorded and hourly averaged PPFD (I_0_) was modified for greenhouse structure transmission losses and used to calculate the mean intercepted PPFD_*i,d*_ for each measured leaf during the last *d* days.


(11) P⁢P⁢F⁢Di,d⁢¯=∑t-1t-dI0(t)exp(-kLAIt,i(t)′)/n

with crop diffuse extinction coefficient *k* (0.72, Heuvelink, 1996) and back extrapolated LAI_*t*_,_*i*_ starting from the end of the previous day to *d* days backward with a total of *n* daylight hours. Note that the specific value of *d* is estimated during parameter estimation.

Leaf photosynthesis assessments on marked leaves started on 5th of April that was 42 days after transplanting. Three non-neighboring plants, located in the center of the greenhouse, were selected for measuring CO_2_ response curves in different vertical canopy levels (1–8). Weekly measurements of photosynthesis CO_2_-response curves (A-C_*i*_ curves, LI-COR 6400; LI-COR Inc., Lincoln, NE, United States) were performed on three plants for all leaves with a length of >10 cm starting with leaf number 9 and terminating with leaf number 39. This corresponded to a leaf-age range from 20 to 57 days at the end of the measurements. All A/C_*i*_ curves were obtained on one of the two-second leaflets of each leaf (counted from petiole-base). Leaf temperature was set at 25°C, and CO_2_ concentration (C_*a*_) was changed stepwise to 400, 350, 300, 300, 250, 200, 100, 400, 450, 500, 550, 600, 800, and 1,000 μmol mol^–1^ while keeping PPFD constant at 1,500 μmol m^–2^s^–1^ at an average leaf vapor pressure deficit of about 2.5 kPa. Several measurements were taken within a period of 10 s and averaged after fluxes had been either stabilized or the maximum measurement time of 120 s was encountered. For obtaining the main biochemical parameters of the FCB model (i.e., *V*_cmax_, *J*_max_ at 25°C) from gas exchange measurements, the fitting approach proposed by Ethier and Livingston (2004) was applied, which implicitly accounts partly for the mesophyll conductance effect. Notably, 2–3 single FCB estimates of *V*_cmax_ and *J*_max_ per layer and date were averaged.

**FIGURE 1:**
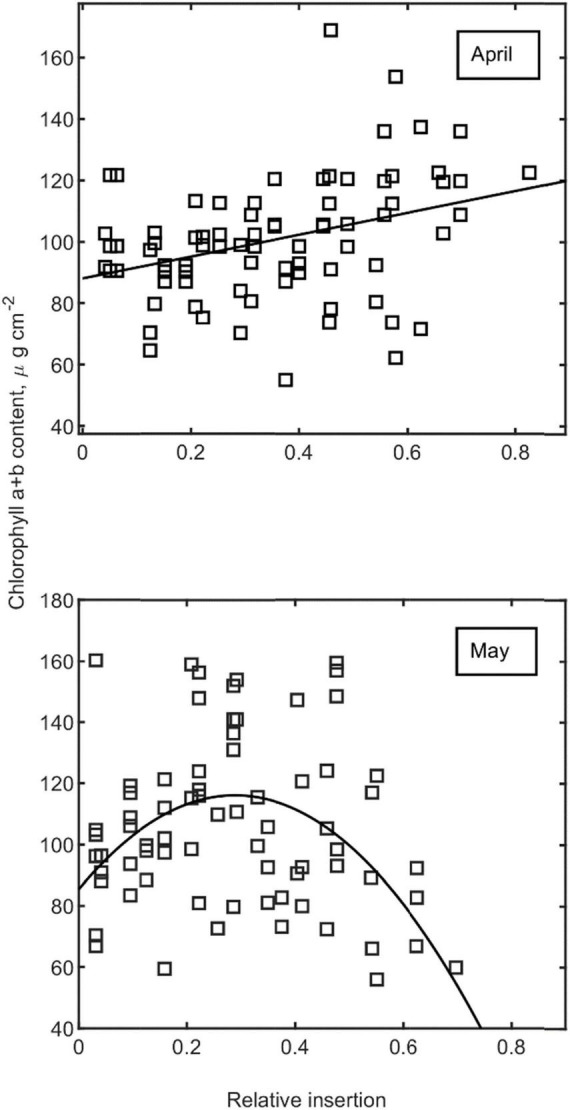
Estimated vertical profiles of chlorophyll content as a function of relative insertion (top leaves = 1, bottom leaves = 0) for the months of April and May. Single estimates (symbols) with linear and quadratic regression functions for April and May, respectively.

A handheld spectrophotometer device (Pigment Analyzer PA-1101, CP, Falkensee, Germany), which measures spectral remission between 320 and 1,120 nm at a spectral resolution (SR) of 3.3 nm (Kläring and Zude, 2009), was used on the same plants and leaves (upper side) as used for gas exchange measurements. We applied the Angers optical dataset (Jacquemoud et al., 2003, SR = 1 nm, dicot leaves) to calibrate the optical output of the Pigment Analyzer according to the following equation:


(12) $$C\,h\,l\,\,\left(\frac{\mu \,g}{c\,m^{2}}\right)=57.74\,\frac{R\,713-R\,709}{R\,703-R\,699}-18.11\;$$

with estimated total chlorophyll (a + b) content per leaf area and measured remissions (of reflectance) (R*) at wavelengths 713, 709, 703, and 699 nm. For calibration (R^2^ = 0.955, *n* = 204), only non-senesced leaves were selected from the dataset while accounting for different SRs between the reference dataset and the device. For *noise* reduction, we only estimated the mean functions of *Chl* with the relative insertion level (bottom leaves = 0) for April and May (*robust linear regression with bisquare weights*, *robustfit procedure*, MATLAB 2020a, refer to Figure 1).

Estimated mean *Chl*(z) profile functions (Figure 1) were further modified by the received sum of PPFD_*i*_ (mol m^–2^) during expansion (21 days) of each leaf *i*, where the effect was assumed to decrease linearly to zero down to an insertion level (*z*_*i*_) of *z*_*i*_ = 0.5, i.e., this initial enhancing effect was assumed to be fully diminished for the lower half of the canopy.


(13) $$C\,h\,l_{i}^{{}^{\prime }}\,\left(z_{i}\right)=C\,h\,l\,\left(z_{i}\right)+m\,\left(P\,P\,F\,D_{i}-\bar{P\,P\,F\,D}\right)\,\,2\,\max\,\left(0,-0.5+z_{i}\right)$$

The coefficient (*m* = 0.0447) was estimated from a regression of corresponding data presented by Trouwborst et al. (2011a) and assumed to apply in an additive manner to the mean profiles in Equation 13. $\bar{P\,P\,F\,D}$ denotes the mean intercepted PPFD during April and May accordingly, while Chl(z_*i*_) stands for the expected mean Chl content computed from relative leaf insertion level alone (mean curves in Figure 1).

**FIGURE 2:**
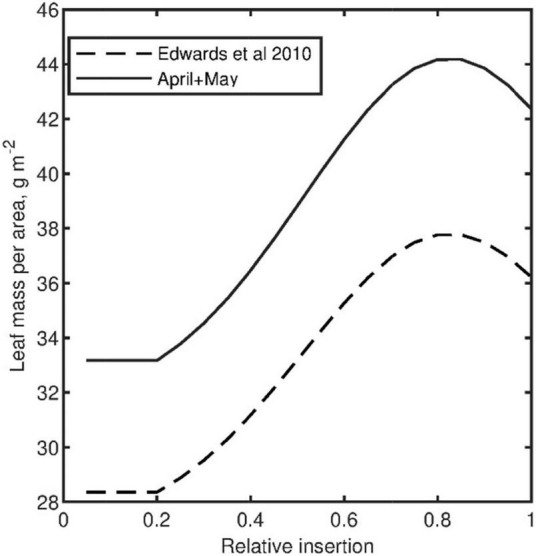
Adopted vertical profile function of leaf mass per area (LMA) (without starch) during the tomato experiment based on LMA measurements of bottom leaves (relative insertion = 0) and an empirical equation form parameterized from Edwards et al. (2010).

The vertical profile of *LMA*, which is also required to estimate leaf optical parameters, was described as an empirical function of leaf position, total leaf number, and bottom value of *LMA* from a reanalysis of functions provided by Edwards et al. (2010) (Figure 2). Specifically, we considered starch as a source of variation in *LMA* that does not add useful information for leaf optical properties modeling. Therefore, a starch-free *LMA* profile was parameterized from a set of published expressions for two cultivars and several months (Edwards et al., 2010). Average *LMA* values obtained at the end of the experiment over the whole canopy were compared well with the calculated mean *LMA* over the adopted *LMA* profile function.

#### Photosynthetic Capacity in Spinach, Eucalyptus, and Cucumber

The profiles of photosynthetic capacity were analyzed with published data of three different crops, i.e., spinach (*Spinacia oleracea*), eucalyptus (*Eucalyptus pauciflora*), and cucumber (*Cucumis sativus*).

Photosynthetic capacity vs. cumulative chlorophyll content for *S. oleracea* and vertical *E. pauciflora* leaves were obtained from Nishio et al. (1993) and Evans and Vogelmann (2006), respectively. The effective extinction coefficient *k* was estimated through Gaussian process regression functions using leaf features *Chl*, *LMA*, and *Car* (refer to Table 2). The measured relative capacity profiles [C_*n*_(c)] were then compared to a normalized form of Equation 7, with estimated φ, *p*_1_*k*_*a*_, and I*.


(14) Cn(c)=φIa(c,wu,g,I*,k)′/φIa(0,wu,g,I*,k)′

Measured properties of horizontal cucumber leaves and photosynthetic light response 7 days after a step change in growth irradiance at 4 different light transitions were tested (Table 1; Trouwborst et al., 2011b). The provided values of *J*_max_ and net photosynthesis rates *A*_*n*_ at 25°C were converted to leaf electron transfer rates *J*_*leaf*_, assuming 50% reduction of dark respiration (*R*_*d*_) in light,


(15) $$J_{L\,e\,a\,f}=\frac{\left(A_{n}+0.5\,R_{d}\right)\,\left(4.5\,C_{i}+10.5\,{\text{Γ}}_{*}\right)}{\left(1-{\text{Γ}}_{*}/C\right)\,C_{i}}$$

**TABLE 1:** Leaf properties used for model testing of photosynthetic capacity profile and electron transport rates.

Species	PPFD μmol m^–2^ s^–1^	*Chl* μg cm^–2^	*LMA* g m^–2^	*Chl2Car*	*w*_*u,g*_
*Spinacia o.^1^*	800	56.3	48	4.46	0.9
	200	48.8	37	4.84	0.9
*Eucalyptus p. ^2^*	Natural	44.8	240	4.25	0.5
*Cucumis s. ^3^*	200→200	57	27.6	5.3	0.9
	50→200	54.9	24.3	5.4	0.9
	200→50	56.3	23.3	5.4	0.9
	50→50	40.0	15.4	5.5	0.9

*^1^Nishio et al. (1993); ^2^Evans and Vogelmann (2006); ^3^Trouwborst et al. (2011b). Chl, chlorophyll a + b; LMA, Leaf mass area; Chl2Car, chlorophyll to carotenoid ratio; w_u,g_, fractional light interception at upper leaf side.*

**TABLE 2:** Parameter estimates for the fit of Equation 6 to electron transport rate of differently light acclimated cucumber leaves.

Parameter	*p*_*i1*_	*p*_*i2*_	θ	*J*_*c,max,mn*_
Unit	−	−	−	mmol e^–^ (mol Chl) ^–1^ s^–1^
Value (CI)	1.51 (1.4–1.6)	0.446 (0.36–0.53)	0.962 (0.93–0.99)	161 (150–174)

*Seven days after step change in light intensity. w_u,g_ = 0.9 (assumed), p_3_ = 0.54, RMSE = 4.22, n = 20. CI: p = 5% confidence interval.*

with CO_2_ compensation point Γ_*_ set to 42.75 ppm (Bernacchi et al., 2001) and leaf internal CO_2_ concentration *C*_*i*_ (ppm).

## Results

### Empirical Description of Simplified Leaf Radiation Transfer Parameters

**FIGURE 3:**
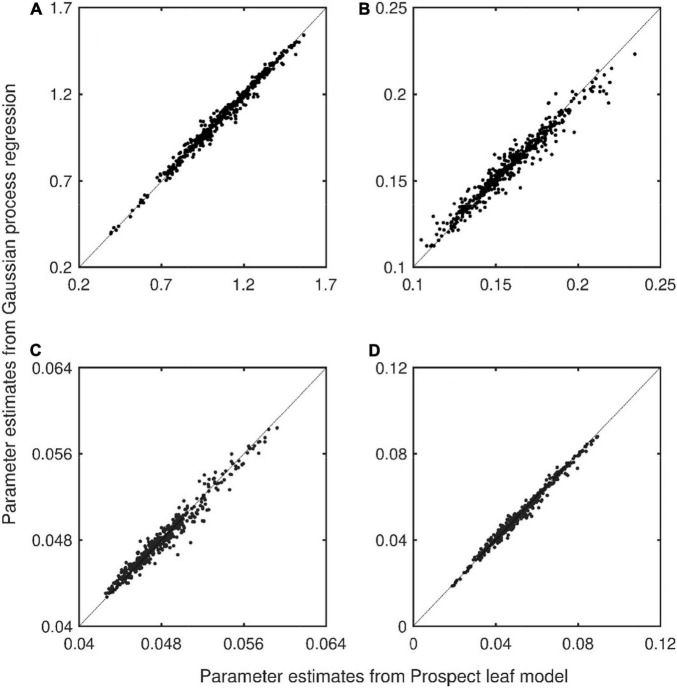
Estimated parameters from the Prospect model (using Equations 1–4) vs. empirical regression estimates using Gaussian process regression *y* = f(*Chl, LMA, Car*) with 1:1 line. *n* = 470, Lopex and Angers leaf optical properties datasets, **(A)** scaling parameter *p*_1_, **(B)** effective leaf extinction coefficient *k*, **(C)** absorption coefficient *k*_*a*_, and **(D)** the product of *p*_1_ and *k*_*a*_. Optical depth is the chlorophyll content (*Chl*; μg/cm^2^).

A major prerequisite for the following analysis is the validity of Equation 1 with profile parameters estimated from bulk leaf properties *Chl*, *Car*, and *LMA*. Setting the coefficient *p*_2_ to 0.664 for all leaves improved the fit of Equation 1 to computed profiles of *I*_*d*_(c) + *I*_*u*_(c) (Equations 3, 4). The root mean squared error (RMSE) decreased from 0.0263 with *p*_2_ = 1 (i.e., the standard approach) to an RMSE of 0.01 (*p*_2_ = 0.664). Figure 3 shows that the remaining parameters (*p*_1_, *k*, and *k*_*a*_) can be fairly well predicted from leaf properties *Chl*, *Car*, and *LMA* using Gaussian process regression. Due to the two-stream nature of radiation transfer and manifested by the *p*_1_ parameter, radiation intensities may exceed 1 (Figure 3A). It is more feasible to estimate the product p_1_k_*a*_ (Figure 3D) than its terms separately.

### Testing for the Coincidence of Photosynthetic Capacity and Light Absorption

**FIGURE 4:**
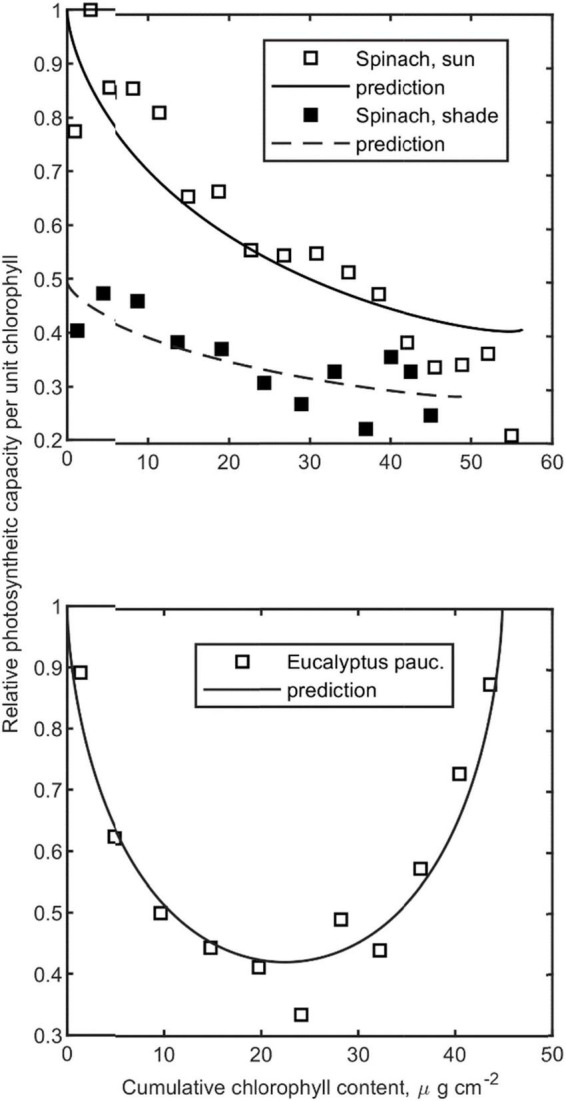
Relative photosynthetic capacity vs. cumulative *Chl*. **(Upper graph)** Sun [*k* = 0.1457, fitted *p*_3_ = 0.53 (0.458–0.628)] and shade [*k* = 0.1455, fitted *p*_3_ = 0.36 (0.156–0.559)]-treated horizontal spinach leaves (w_*u,g*_ = 0.9, Nishio et al., 1993). **(Lower graph)** Vertical isobilateral leaves of E. *pauciflora* (*k* = 0.2535, fitted *p*_3_ = 0.73 (0.675–0.789), w_*u,g*_ = 0.5, Evans and Vogelmann, 2006). Confidence limits are given in parenthesis, *p* = 0.05.

To test Equation 7, we compared the profiles of the normalized light gradient *I*_*a*_ (*c*, *w*_*u*,*g*_, I_*_, *k p*_3_)/*I*_*a*_ (0, *w*_*u*,*g*_, I_*_, *k p*_3_) with published profiles of maximum photosynthetic capacity in Spinach (Nishio et al., 1993; Terashima et al., 2009) and *E. pauciflora* (Evans and Vogelmann, 2006; Figure 4). While estimating *k* from given values of *Chl*, *Car*, and *LMA*, we could not justify a perfect match between light absorption and capacity profiles as fitted *p*_3_ was always significantly lower than one [5% confidence region for all fitted *p*_3_ = (0.156, 0.789)]. As those datasets are most suitable for the identification of *p*_3_, we set it in the following to the mean of the obtained 3 estimates (*p*_3_ = 0.54).

### Testing Modified Electron Transfer by Light Acclimation in Cucumber

**FIGURE 5:**
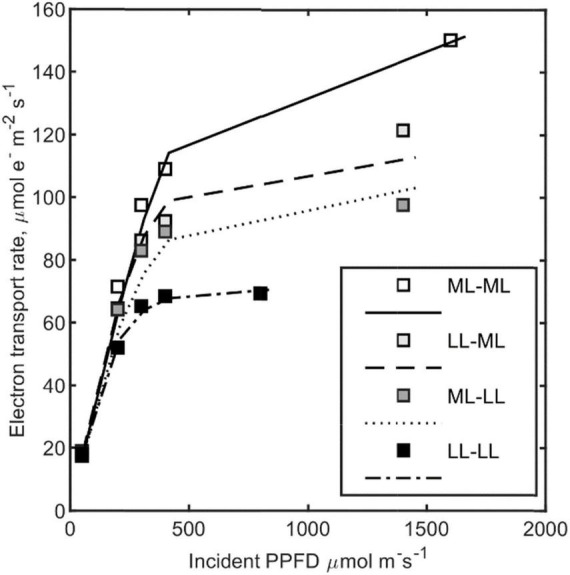
Measured electron transport rates (symbols) and predictions by Equation 6 (lines) vs. incident photosynthetic photon flux density (PPFD) at 7 days after a step change in light intensity for cucumber leaves (Trouwborst et al., 2011b), ML-ML (I_1_:200 → I_2_:200 μmol m^2^ s^–1^), LL-ML (I_1_:50 → I_2_:200 μmol m^2^ s^–1^), LL-ML (I_1_:200 → I_2_:50 μmol m^–2^ s^–1^), and LL-LL (I_1_:50 → I_2_:50 μmol m^–2^ s^–1^). Plants were measured after 7 days growing at intensity I_2_. Lines connect computations at assessed PPFD (i.e., symbols). *n* = 20, root mean squared error = 4.22 μmol e^–^ m^–2^ s^–1^.

Published data for electron transport of cucumber leaves (Trouwborst et al., 2011b) could be predicted with fitting parameters to Equation 5 (Table 2 and Figure 5). The estimated empirical model for I* is as follows:


(16) $$I^{*}=p_{i\,1}\,\left(p_{i\,2}\,I_{1}+\left(1-p_{i\,2}\right)\,I_{2}\right)$$

With *p*_*i2*_ being significantly greater than zero (Table 2), a large influence exists from the preceding light intensity prior to step change. Note that calculated I* is here greater than the mean intensity during growth.

The minimum of *J*_*c,max*_ (*J*_*c,max,mn*_) was only active at constant low light treatment (LL-LL). The estimated value for θ (0.962) will also be used in subsequent steps.

### V_*cmax*_ and J_*max*_ in Different Canopy Levels and Leaf Ages in a Tomato Crop

#### Parameter Estimation

**FIGURE 6:**
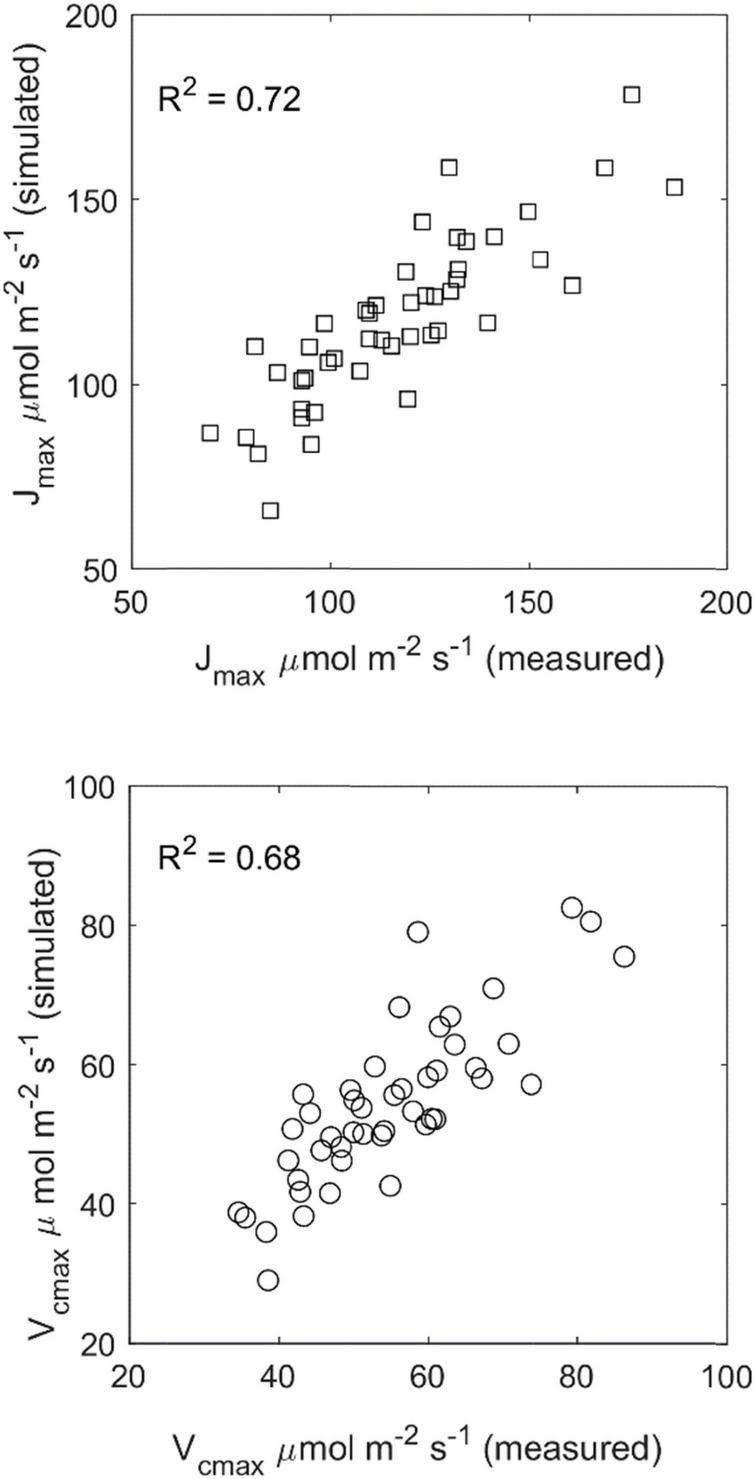
Comparison of predicted vs. measured maximum rates of carboxylation (*V*_*cmax*_) and electron transport (*J*_max_) at different canopy depths over 2 months in a tomato canopy. *n* = 46. Refer to Table 2 for more details.

Overall, the tested mechanistic model for photosynthetic light acclimation proved to be successful (Figure 6). The model could explain 68 or 72% of the observed variance for *V*_cmax_ and *J*_max_, respectively (Table 3). The estimated empirical model for I* is as follows:


(17) $$I^{*}=p_{i\,1}\,\bar{P\,P\,F\,D_{i,d}\,}$$

**TABLE 3:** Parameter estimates for the fit of Equations 5, 10 to measure *J*_*max*_ and *V*_*cmax*_ in tomato.

Parameter	*p*_*i1*_	*p*_4_	*p*_*J0*_	*p*_*J1*_
Unit	−	−	mmol e^–^ (mol *Chl*) ^–1^ s^–1^	mmol e^–^ (mol *Chl*) ^–1^ s^–1^h^–1^
Value CI	0.586 (0.54–0.63)	0.437 (0.41–0.47)	304 (273–336)	7.00 (5.8–8.1)

*w_u,g_ = 0.7, p_3_ = 0.54, φ = 0.468, θ = 0.962, RMSE-J_max_ = 13.87, n = 46, RMSE-V_cmax_ = 7.02, n = 46. CI = 5% confidence intervals.*

Best fitting results (in terms of the sum of squares) were obtained manually with *d* = 3, e.g., 3 previous days were used to compute $\bar{P\,P\,F\,D_{i,d}}$ for each leaf (equally weighted mean calculation). Alternative non-linear time weighting schemes improved the model fit marginally toward *d* values of 4–5 days.

The proportionality constant *p*_*i1*_ could be well identified for this dataset but at a lower value compared to cucumber (Table 2).

For the time dependence of minimum and maximum *J*_*c,max*_ (*J*_*c,max,mn*_, *J*_*c,max,mx*_), which is here considered an aging process, the following relation was adopted.


(18) $$J_{c,m\,a\,x,m\,n}=p_{J\,0}+p_{J\,1}\,P\,R_{s\,u\,m}^{0.5}\;J_{c,m\,a\,x,m\,x}=n\,\,J_{c,m\,a\,x,m\,n}$$

with an hourly sum of the phenology response since leaf appearance *PR*_*sum*_ and empirical parameters *p*_*J0*_ and *p*_*J1*_. The factor *n* was set to 2.6, the mean ratio obtained from experimental estimates (Evans and Seemann, 1989) on bulk leaves of several species.

For about 46% of the tested leaves, the photosynthetic capacity was constrained by *PR*_*sum*_, i.e., *J*_*c,max,mn*_(t) was set as a lower limit in Equation 7.

#### Model Simulation

**FIGURE 7:**
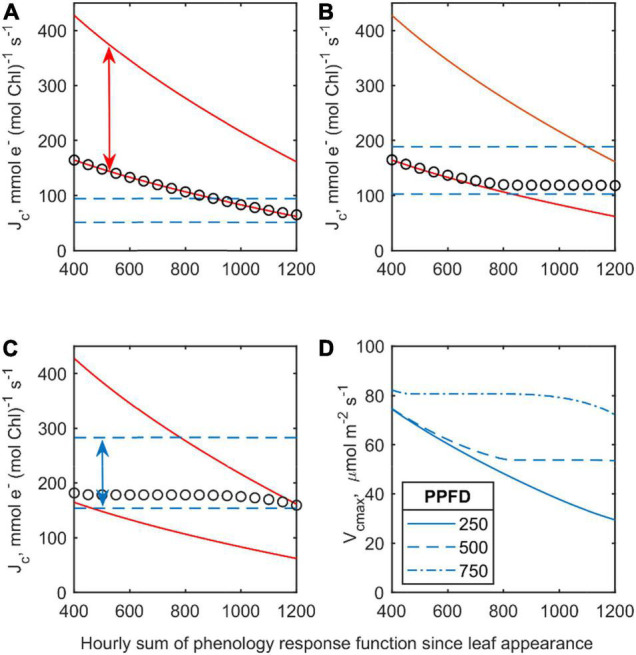
**(A–C)** Simulated adaptation ranges of electron flow (lines) and final mean leaf rate (symbols) over time at different time constant PPFD: **(A)** 250 μmol m^–2^ s^–1^, **(B)** 500 μmol m^–2^ s^–1^, and **(C)** 750 μmol m^–2^ s^–1^. Lower and upper red lines are *J*_*x,max,mn*_ and *J*_*x,max,mx*_, respectively. Lower and upper blue dashed lines are minima and maxima set by the profile of absorbed light, respectively. **(D)** Corresponding calculated rates of *V*_cmax_ over time.

Assuming constant leaf properties and light intensities, different limitation onsets of electron flow by aging and light adaptation were investigated. At low light intensities (PPFD = 250 μmol m**^–^**^2^ s**^–^**^1^, Figure 7A) the computed mean rate of electron transfer (symbols in Figures 7A–C) was almost entirely determined by the ontogenetic prescribed lower limit of electron transfer which decreases monotonically over time. Similarly, the calculated *V*_*cmax*_ (Figure 7D) was decreasing continuously over time. In contrast, at higher PPFD (750 μmol m**^–^**^2^ s**^–^**^1^, Figure 7C), the electron flow could be determined by (constant) light acclimation and was later constrained by the upper limit of the ontogenetic prescribed range of electron flow (Figure 4C). This scenario results in an almost time-invariant behavior of *V*_*cmax*_ (Figure 4D).

## Discussion

### Model-Framework Validity

We present a novel mathematical framework (Equations 2, 5–10) to describe the time dependency of the FCB photosynthetic model parameters (φ′, θ, *J*_*max*_, and *V*_*cmax*_) caused by progressing leaf phenology and light acclimation. The derived relations build on previous work to model light acclimation (Badeck, 1995) or whole leaf electron transport rates (Buckley and Farquhar, 2004). The proposed model framework requires an accurate specification of the incoming radiation field [PPFD(t), w_*u,m*_, and w_*u,g*_], additional leaf traits (*Chl*, *Car*, and *LMA*), and further parameters (*J*_*c,max,mx*_, *J*_*c,max,mn*_, I*) that are likely functions of perceived temperatures and intercepted light intensities during leaf growth (Equations 16–18).

We tested the capability of the framework to predict published intra-leaf photosynthetic capacity profiles (Figure 4), light response curves for differently light-adapted cucumber leaves (Figure 5), and measured *J*_*max*_ and *V*_*cmax*_ values at different times and canopy depths in a tomato crop. To limit the degree of freedom for each step, we estimated several parameters hierarchically from independent datasets, e.g., *p*_1_*k*_*a*_ and *k* using generated leaf optics data, *p*_3_ from capacity profiles, and θ from light response curves.

Clearly, to explore the full validity of our proposed theory, more experimental work with vertically and horizontally grown tomato and cucumber crops is required. An evident key role in this matter was identified in leaf *Chl* content. Being an integration variable (e.g., Equation 10) it also influences intra-leaf absorption parameters *via* Gaussian process regression. This fits well with recent observations in various species of *V*_*cmax*_ and *J*_*max*_-*Chl* relations being better predictors than leaf nitrogen (Qian et al., 2021). However, neither its repeatable measurement nor its empirical prediction of *Chl* in time and space seems to be trivial. For tomato, *Chl* is dependent on the received light intensity during leaf expansion (Equation 13), Trouwborst et al. (2011a), while it declines with canopy depth (Figure 1).

### Model Framework in a Current Scientific Context

Due to multiple and internal reflections (*r*_*i*_) at the leaf epidermis-air interface (Equation 3), the total received irradiance at the topmost mesophyll layer may exceed the incident intensity (Figure 3). This phenomenon has been theoretically predicted and measured (Vogelmann and Björn, 1984). Therefore, the specific parameter *p*_1_ was introduced (Figure 3A). A more effective way to predict the profile of absorbed radiation (Equation 2); however, is combining *p*_1_ with *k*_*a*_, i.e., *p*_1_*k*_*a*_ (Figure 4D).

Analogously to the distribution of leaf photosynthetic capacity and leaf nitrogen content with canopy depth, a covariation of photosynthetic capacity profiles with intra-leaf absorbed radiation was observed (Figure 4). Consistently over all three observed capacity profiles, the agreement was imperfect: *p*_3_ (on average 0.54) was significantly lower than 1. Earlier studies with whole leaves support our finding: A canopy scale meta-study estimated an analog reduction of the light extinction coefficient by 0.5 (Hikosaka et al., 2016).

The obtained estimate for θ = 0.962 for cucumber leaves (Table 2) corresponds well with an average figure of 0.965 reported by Terashima and Saeki (1985) for chloroplast and cell suspensions. Similarly, *J*_*c,max,mn*_ estimated at 161 was similar to measurements in shaded cucumber leaves of 160 (PPFD = 120 μmol m^–2^ s^–1^; Evans, 1989). For dicot plants common bean (*Phaseolus vulgaris*) and tobacco (*Nicotiana tabacum*), there is strong evidence that the ratio of the leaf cytochrome b_6_f complex to chlorophyll content is the major target for both light acclimation and leaf aging (Schöttler and Tóth, 2014), which is linear related to electron flow (Evans and Seemann, 1989). Moreover, this ratio changes for tobacco by a factor of 2.45 from low to high light-adapted leaves (Schöttler and Tóth, 2014), which is close to the adopted value *J*_*c,max,mx*_/*J*_*c,max,mn*_ = 2.6 (Evans and Seemann, 1989) based on measured electron transport rates.

A strong correlation between *V*_*cmax*_ and *J*_*max*_ is well known. Wullschleger (1993) presented a *V*_*cmax*_ to *J*_*max*_ ratio of 0.431 for vegetable crops (17 species), obtained from A/C_*i*_ curves assuming implicitly a fixed curvature θ of leaf electron transfer. This ratio evolves automatically as parameter *p*_4_ in Equation 10, with an estimated value of p_4_ = 0.437 for tomato (Table 3), a remarkable agreement of Wullschleger’s result and our estimate.

The bifacial nature of leaf morphology of dicot plants is often accompanied by different leaf reflectance and transmittances measured from the adaxial and abaxial leaf sides (De Lucia et al., 1991; Stuckens et al., 2009). This indicates different effective two-stream parameters depending on whether light is incident on the adaxial or abaxial leaf side. Therefore, additional research would be needed to investigate the necessity of introducing different parameters for the palisade and spongy mesophyll layers (Terashima et al., 2009). Especially for cases with significant light incidence from the lower leaf side, either during acclimation or measurement, this might be of importance.

### Extensions to the Model Framework

The major foundation of this analysis is the assumption of the validity of the two-stream approximation of radiation transfer for leaves. This includes the need for identifying two parameters (*k*_*s*_, *k*_*a*_) from total leaf transmittance and reflectance while accounting for diffuse Fresnel reflectance/transmittances at the leaf boundaries (Equations 3, 4). This approach assumes perfectly diffused radiation streams, with equal probability of backward and forward scattering of photons, setting the anisotropy parameter for scattering *g* to zero. However, an accurate approximation to the radiation transfer equation for a scattering and absorbing slab was recently derived (Liemert et al., 2019). This solution could be a useful asset in improving parameter calibration of Equation 2 or similar functions, which eventually can lead to the derivation of better approximations; even different incidence angles and refraction index changes at the leaf surface can be accounted for Liemert et al. (2019). For that, an independent spectral parameterization of the anisotropy parameter g(λ) (or the scattering phase function) would be required. Measurements on various biological tissues indicate a rather smooth and slow change of g(λ) over the visible wavelength range (Jacques, 2013).

### Ways for Practical Application

Both from a theoretical and experimental standpoint, the quantification of received radiation fluxes per leaf (patch) within plant canopies is not straightforward. In real (commercial cultivated) canopies, the leaf-specific and time-dependent estimation could be supported with imaging techniques. One solution would be the combination of a hemispherical gap fractions distribution from fisheye imaging (Eichelmann et al., 2005) with our model framework. Model predictions and accurate specification of the incoming radiation field could be a basis for a powerful monitoring tool in vertical crop stands. In addition, there is a growing number of functional structural plant model (FSPM) codes (Buck-Sorlin et al., 2011; Sarlikioti et al., 2011) and libraries (Bailey, 2019), which are in principle well suited to provide this information even on a leaf patch basis in virtual canopies.

## Conclusion

In this study, we extended a previous leaf model for electron transport rate (Buckley and Farquhar, 2004) to account for the phenomenon of non-perfect acclimation of photosynthetic capacity to absorbed radiation within the mesophyll. Adopting the two-stream solution of radiation transfer with cumulative chlorophyll content, we derive the scattering and absorption coefficients from the total reflectance and transmittance of leaves. This allowed the derivation of an improved simplified model for absorbed radiation profile and corresponding lumped parameters, which can be estimated just from total chlorophyll, carotenoid, and dry mass content per leaf area using machine learning methods. A reanalysis of published datasets with this simplified model revealed a significant derivation of measured photosynthetic capacity profiles from calculated absorption profiles, while this deviation can be resolved empirically.

Furthermore, the applicability of the modified model was tested on light acclimation on published experimental data with cucumber (Trouwborst et al., 2011b) and with a self-performed tomato cultivation experiment. These tests revealed that ontogenetic constraints are likely to be superimposed on light intensity effects within the leaf mesophyll.

## Data Availability Statement

The raw data supporting the conclusions of this article will be made available by the authors, without undue reservation.

## Author Contributions

JG: model development, model conception and realization, manuscript writing, data management, simulations, and figures. WY: experimental measurements, first draft of manuscript, and model conception. OK: experimental design, experimental supervision, and manuscript writing. All authors contributed to the article and approved the submitted version.

## Conflict of Interest

The authors declare that the research was conducted in the absence of any commercial or financial relationships that could be construed as a potential conflict of interest.

## Publisher’s Note

All claims expressed in this article are solely those of the authors and do not necessarily represent those of their affiliated organizations, or those of the publisher, the editors and the reviewers. Any product that may be evaluated in this article, or claim that may be made by its manufacturer, is not guaranteed or endorsed by the publisher.
